# Efficacy of Gastrointestinal Endoscopy in 398 Patients With Iron Deficiency Anemia Who Lack Gastrointestinal Symptoms: Basrah Experience

**DOI:** 10.7759/cureus.9206

**Published:** 2020-07-15

**Authors:** Samih A Odhaib, Miaad J Mohammed, Saad Hammadi

**Affiliations:** 1 Adult Endocrinology, Faiha Specialized Diabetes, Endocrine and Metabolism Center, University of Basrah College of Medicine, Basrah, IRQ; 2 Diagnostic Radiology, Al-Refaee General Hospital. Thi-Qar Health Directorate, Thi-Qar, IRQ; 3 Internal Medicine, University of Basrah College of Medicine, Basrah, IRQ

**Keywords:** iron deficiency anemia, gastrointestinal endoscopy, asymptomatic, malignancy, celiac disease, basrah

## Abstract

Introduction

The diagnosis of iron deficiency anemia (IDA) relies heavily on symptom presentation, and patients lacking typical gastrointestinal (GI) symptoms represent a diagnostic challenge. IDA may be the initial manifestation of underlying pathology. This study sought to evaluate the effectiveness of different GI endoscopic studies in patients with IDA who lack GI symptoms.

Methods

We conducted an observational, multicenter retrospective analysis of 398 asymptomatic IDA patients admitted for GI endoscopic diagnosis from 2006 to 2016. Baseline measurements included hemoglobin, serum ferritin, mean corpuscular volume, serum iron, total iron-binding capacity, and transferrin saturation. We analyzed demographic characteristics, duration of hospital stay, the degree of severity of anemia, and endoscopic findings.

Results

The mean age of the study population was 52±9 years (range, 23 to 85 years), and 53% were men. Most patients were older than 45 years (n=353, 89%) with mild to moderate IDA. Patients underwent esophagogastroduodenoscopy (EGD, n=102), colonoscopy (n=271), or bidirectional endoscopy (n=25). The mean hospital stay was 2.72±1.66 days. The most common EGD results were atrophic gastritis (n=31), peptic ulcer (n=25), and negative findings (n=25). The most common colonoscopic results were negative findings (n=118), nonspecific colonic inflammatory changes (n=117), and non-bleeding hemorrhoids (n=29). We found no significant association between any endoscopic findings and age, gender, the severity of anemia, and length of hospitalization.

Conclusions

The presence of symptoms is of limited value in guiding diagnostic procedures concerning GI etiologies. Asymptomatic patients with IDA patients should receive an endoscopic examination irrespective of iron parameters, age, or gender for potentially treatable pathologies, especially for patients with suspected malignancies.

## Introduction

Historically, there exists no substantial evidence for performing gastrointestinal (GI) endoscopic evaluation in young patients with iron deficiency anemia (IDA) who lack GI symptoms. Endoscopic GI lesions, including colorectal carcinoma (CRC), villous adenoma, inflammatory bowel disease (IBD), and gastric ulcer, are common in this group of patients. Investigating asymptomatic patients for such possibilities is of pivotal importance, given patient age is the most reliable predictor for GI pathology [1,2]. The GI endoscopy, and especially a colonoscopy, may be helpful for identifying clinically significant sources of IDA, even in asymptomatic young patients [1,3]. The concern surrounding the diagnosis of malignancies in this group compels a complete and rigorous GI tract examination [4,5].

Being asymptomatic causes a diagnostic challenge, as the cases may be identified during routine laboratory testing, screening for CRC, or they can present with atypical non-GI concerns [6]. Asymptomatic GI malignancies and celiac disease (CD) may have a different clinical spectrum in the form of IDA, and exclusion of these conditions is crucial - the diagnosis of which will lessen the documented future high mortality of such lesions in this patient population [7,8].

In this study, we tried to evaluate the effectiveness of different GI endoscopic studies in patients who have IDA with no GI symptoms.

## Materials and methods

**Figure 1:**
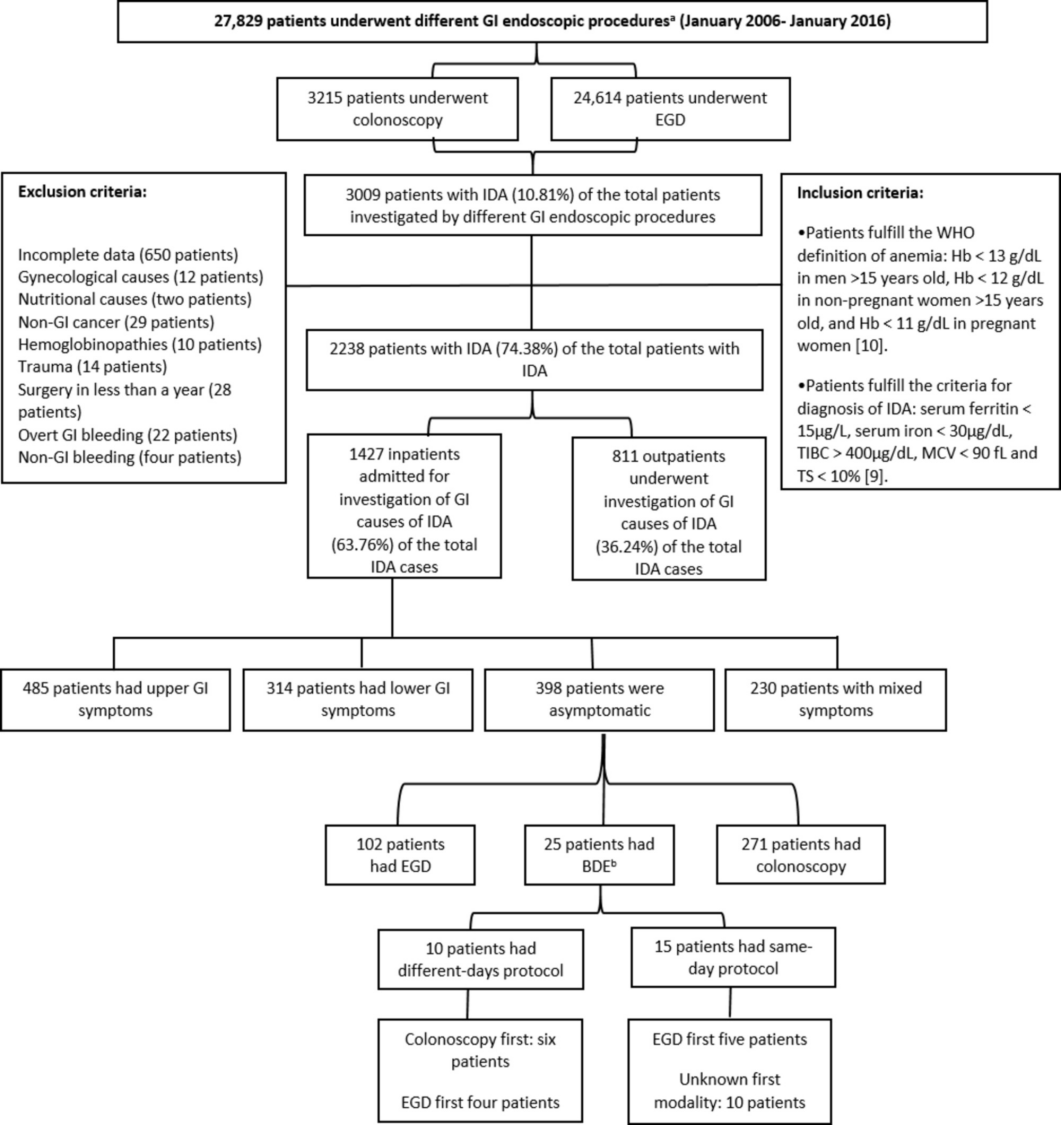
Flow chart of data collection during the study for 398 asymptomatic patients admitted for investigation of the cause of IDA. BDE, bidirectional endoscopy; EGD, esophagogastroduodenoscopy; GI, gastrointestinal; Hb, hemoglobin; IDA, iron deficiency anemia; MCV, mean corpuscular volume; TIBC, total iron-binding capacity; TS, transferrin saturation; WHO, World Health Organization. ^a^All GI endoscopic procedures utilized the Pentax Medical (HOYA Group, Shinjuku, Tokyo) or Olympus (Olympus Surgical Technologies America, Southborough, MA) endoscopic system. ^b^When the EGD and colonoscopy were done on the same day or in different days up to four weeks apart.

This observational study involved the retrospective analysis of the medical records of 398 patients with IDA in the absence of GI symptoms who presented to Al-Sadr and Faihaa Teaching Hospitals, and the Basrah Oncology and Hematology Center for diagnosis of the etiologies of IDA from January 2006 to January 2016. Medical record data collected were not electronic, as the collection period occurred before the advent of automated records. We evaluated data from endoscopy, laboratory, histopathology, and admission units of the three centers. Figure 1 illustrates the process of data collection, the inclusion and exclusion criteria, and the selection of the patients for final analyses [9,10].

All enrolled patients had measurements of their hemoglobin (Hb), serum ferritin, mean corpuscular volume (MCV), serum iron, total iron-binding capacity (TIBC), and transferrin saturation (TS). We tabulated the data according to the following:

• Demographic characteristics.

• Duration of hospitalization.

• Different GI endoscopic findings.

• The severity of IDA [9]:

o Severe IDA when Hb <9 g/L, serum ferritin <9 µg/L, MCV ˂ 70 fL, serum iron ˂ 15 µg/dL, TIBC ˃ 400 µg/dL, TS ˂ 3.5%.

o Mild to moderate IDA when Hb 9-13 g/L, serum ferritin 9-15 µg/L, MCV 70-90 fL, serum iron 15-30 µg/dL, TIBC 360-400 µg/dL, TS 3.5-10%.

The patients were diagnosed with a biopsy-proven CD if an endoscopic biopsy revealed Marsh type 3 (A, B, and C), according to the American College of Gastroenterology clinical guidelines [11]. The main concern focused on type 3, in which there are crypt hyperplasia and increased intraepithelial lymphocytes, along with partial villous atrophy in type 3A, subtotal villous atrophy in type 3B, and total villous atrophy in type 3C.

Data were entered and matched via Microsoft Access and Excel and then analyzed on IBM SPSS Statistics for Windows, version 23.0 (IBM Corp., Armonk, NY). The study used bivariate analysis with the mean±standard deviation or frequency (%) for data expression. The study considered a p<0.05 to be statistically significant.

## Results

Table 1 demonstrate the process of data collection and some demographic characteristics in asymptomatic patients with IDA. There were 398 patients with IDA who lack any GI symptoms on initial presentation. These patients represented 27.9% of the admitted patients with IDA during the study period. The study population had mild to moderately severe IDA (Table 1). There was a slight male preponderance in the study population; the male-to-female ratio was 1.2/1. The mean age of the patient population was 52±9 years (range, 23 to 85 years), with 88.7% of the patients aged ≥ 45 years (n=353).

**Table 1:** The demographic characteristics of 398 asymptomatic inpatients who were admitted for investigation of the cause of iron deficiency anemia. GI, gastrointestinal; IDA, iron deficiency anemia; Ig, immunoglobulin.

Parameters		n
Male gender (%)		211 (53.02)
Mean age (years ± standard deviation)		52±9
Mean duration of hospitalization (days ± standard deviation)		2.72±1.66
Dual lesions		8 (2.01)
Celiac disease diagnosis	Referred for celiac disease diagnosis (%)	53 (13.3)
	Antitissue transglutaminase antibody IgA positive (%)	17 (4.3)
	Antitissue transglutaminase antibody IgG positive (%)	10 (2.5)
	Biopsy-proven celiac disease (%)	15 (3.8)
	Mean age (years ± standard deviation)	41.0±10.0
Malignancies	Upper GI malignancies (%)	16 (4.02)
	Lower GI malignancies (%)	10 (2.5)
IDA parameters	Mean ferritin µg/L	9.94±3.34
	Mean hemoglobin g/L	7.07±2.11
	Mean corpuscular volume fL	62.84±9.76
	Mean serum iron µg/dL	21.78±6.38
	Mean total iron-binding capacity µg/dL	435.04±64.05
	Mean transferrin saturation%	5.17±1.81

**Figure 2:**
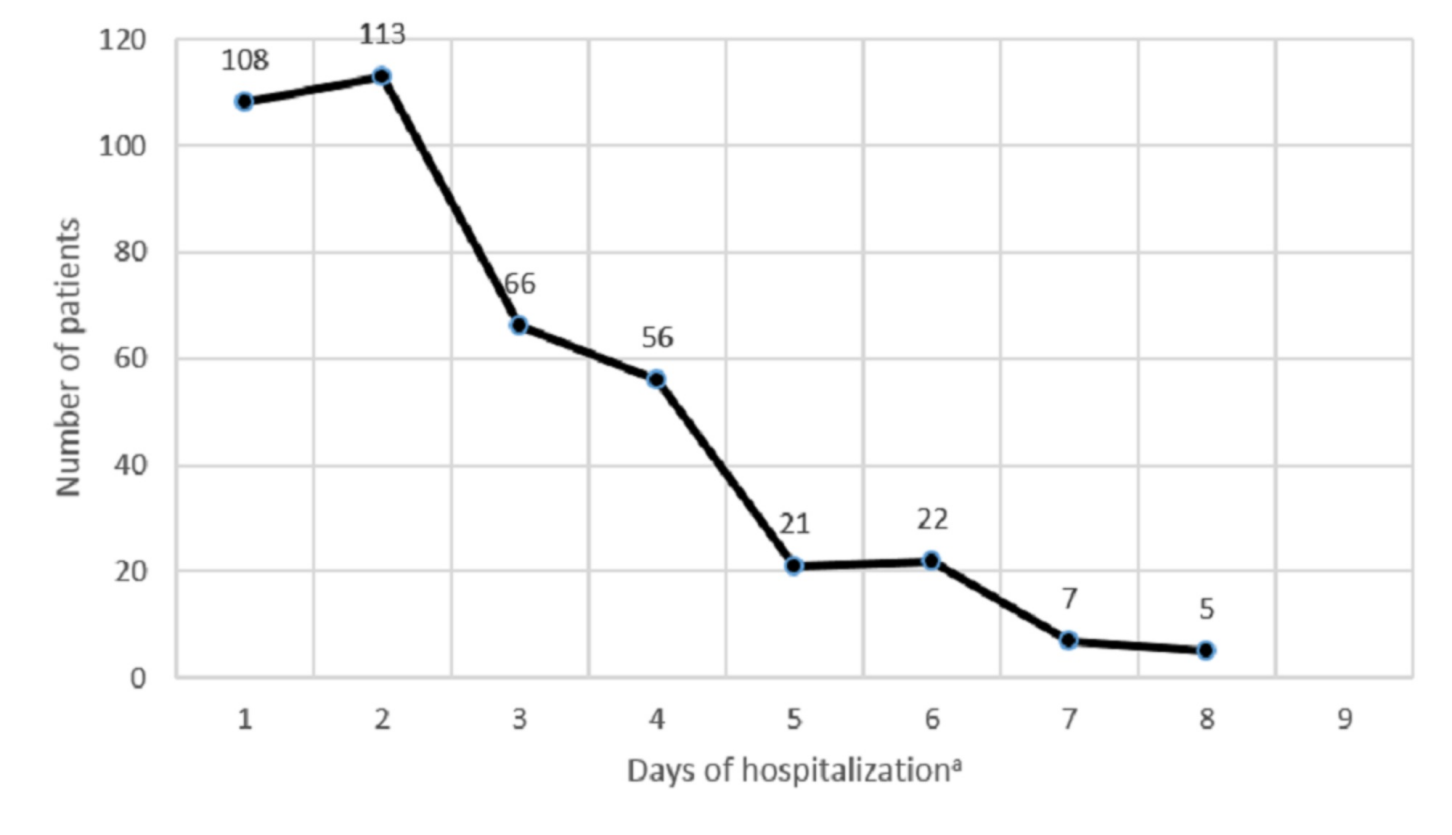
The duration of hospitalization of 398 asymptomatic patients who underwent different gastrointestinal endoscopic procedures for etiological diagnosis of iron deficiency anemia. ^a^There was no association between the duration of hospitalization with the age, gender, severity of iron deficiency anemia, and the endoscopic yield (p>0.05).

The range of hospital length of stay for the asymptomatic inpatients was one to eight days, with a mean duration of 2.72±1.66 days (Figure 2). The hospital stays for patients who received bidirectional endoscopy (BDE) was calculated by combining both admission sessions (if the patient had more than one admission). There was no association between the hospitalization stay with the age, gender, severity of IDA, and the endoscopic yield (p>0.05).

Fifty-three patients were referred for suspected CD, of whom, only 15 patients had biopsy-proven CD (i.e., 3.77% of total asymptomatic inpatients had CD). The mean age for asymptomatic CD patients was 41±10 years (Table 1).

**Figure 3:**
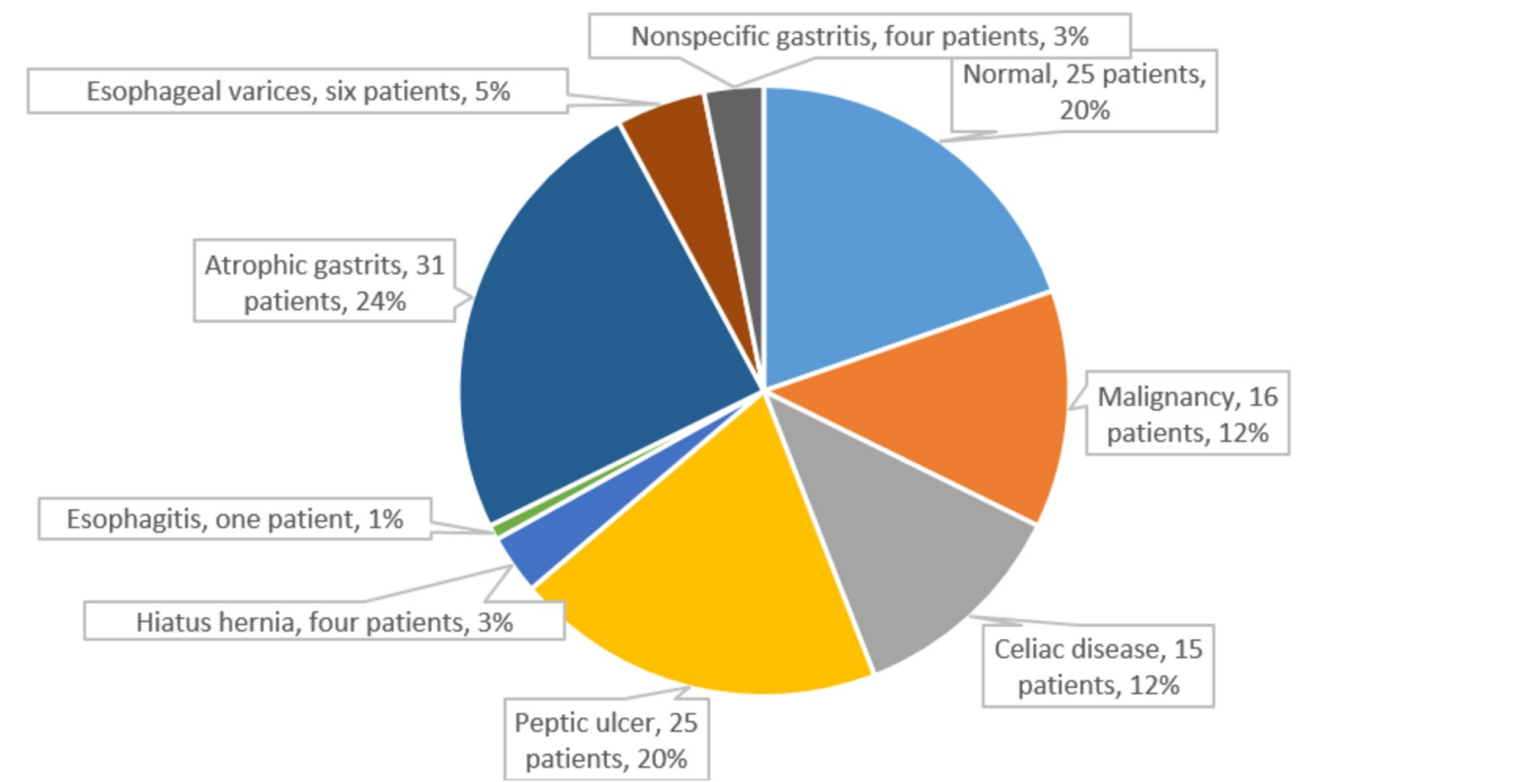
Pie chart of different upper GI endoscopic findings of 127 asymptomatic patients with IDA. The data include 102 patients who underwent EGD as a sole evaluation procedure and 25 patients who underwent EGD as a part of bidirectional endoscopy. The degree of iron deficiency anemia does not correlate with the different yields in EGD (p>0.05). The mean age for patients with upper GI malignancies was 55±6 years (range, 46 to 67 years). EGD, esophagogastroduodenoscopy; GI, gastrointestinal; IDA, iron deficiency anemia.

**Figure 4:**
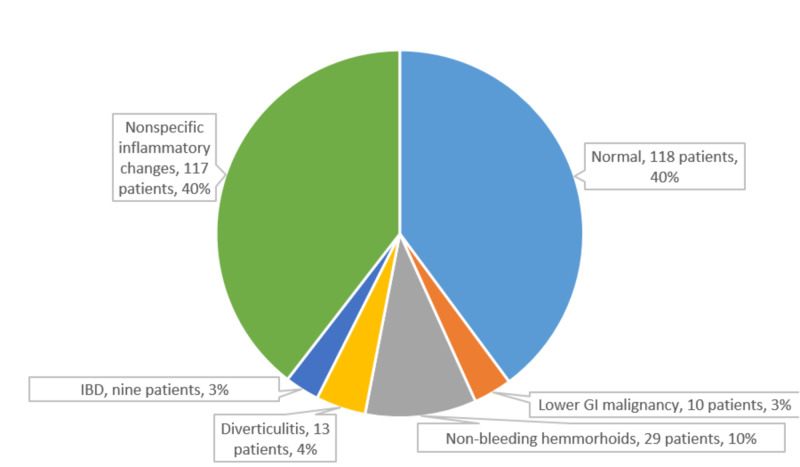
Pie chart of different colonoscopic findings of 296 asymptomatic patients with IDA. The data include 271 patients who underwent colonoscopy as a sole evaluation procedure and 25 patients who underwent colonoscopy as a part of bidirectional endoscopy. The degree of IDA does not correlate with the different yields in lower GI endoscopy (p>0.05). The mean age of patients with lower GI malignancies was 51±8 years (range, 34 to 65 years). IBD, inflammatory bowel disease; GI, gastrointestinal; IDA, iron deficiency anemia.

Figures 3-4 illustrated the different endoscopic findings for asymptomatic patients. The study did not include the negative endoscopy and nonspecific gastritis and inflammatory changes as significant findings.

As shown in Table 2, the study found no significant association between the malignancy diagnosis with the gender, age, or IDA severity in the 26 asymptomatic patients with different GI malignancies.

**Table 2:** The association between the malignancy diagnosis in asymptomatic patients with the age, gender, and different IDA parameters. IDA, iron deficiency anemia.

Parameters	Malignancy diagnosis N=26 (%)	p
Male gender (n=211)	14 (53.85)	0.152
Age ≥ 45 years (n=353)	24 (92.3)	0.799
Serum ferritin ≤9 µg/L (n=212)	14 (53.85)	0.156
Hemoglobin <9 g/L (n=318)	22 (84.62)	0.280
Mean corpuscular volume <70 fL (n=320)	22 (84.62)	0.284
Serum iron <15 µg/dL (n=102)	6 (23.08)	0.460
Total iron binding capacity > 400 µg/dL (n=262)	18 (69.23)	0.245
Transferrin saturation < 3.5% (n=102)	5 (19.23)	0.516

## Discussion

This study sought to address the upper and lower GI endoscopic yield in a cohort of patients with IDA who lack GI symptoms, as the cause of IDA for patients without an etiologic diagnosis remains speculative. IDA may be the initial indicator for a serious undiagnosed, underlying malignancy that may progress silently over time.

IDA, due to occult GI blood loss, usually remains unnoticed until the patient becomes symptomatic (12). Although GI lesions were detected more often in symptomatic patients, asymptomatic patients with IDA may also have considerable GI findings that may or may not be related to the nonspecific symptoms of IDA [4,12-14].

The delay in seeking medical care is considerable, which is why the level of anemia in the enrolled patients was moderate to severe. The blood parameters levels of IDA (Hb, ferritin, iron, MCV, TIBC, and TS) in this study are similar to patients in many studies [4,5,12,14,15]. The retrospective design of this study makes tracking these patients’ delays in seeking medical care difficult.

We found no correlation between the gender, age, and degree of IDA with the endoscopic yield, which was similar to that reported by McIntyre et al. [16]. Previous studies had found that increasing age, male gender, low MCV and ferritin, positive fecal occult blood testing (FOBT), and prior use of nonsteroidal anti-inflammatory drugs (NSAIDs) were useful predictors of relevant endoscopic findings in this group of patients [2,4,12,14].

The nearly equal gender distribution in this study aligns with that of many highly cited international studies reporting a 1:1 male-to-female ratio [4,5,12]. We found no significant relationship between gender and the GI endoscopic findings, especially for GI malignancy. While the current guidelines recommend the same diagnostic protocols in young and older men with IDA, a paucity of data supports this approach [3].

Several previous studies contained patient populations that were disproportionately gendered, such as the study by Annibale et al. [14], who reported GI endoscopic findings in 581 asymptomatic women and 87 men with IDA. Other studies had demonstrated a female predominance, with a male-to-female ratio of 1:1.6 [13,17,18]. Kim et al. evaluated the GI endoscopic findings of young men only, and suggest that endoscopy should be recommended in asymptomatic young men with IDA [1]. Todd et al. concluded that GI referral is not needed in asymptomatic women with IDA younger than age 50 unless they do not respond to adequate iron supplementation and treatment of other identified causes [19].

The mean age of the asymptomatic patients with IDA in this study was 52±9 years, which is a similar mean age in Majid et al. study, which was 52.1±16.8 years [12]. Other international studies had revealed different mean ages, ranging from 39 to 83 years [1,4,15,17,18], with different demographic characteristics and inclusion criteria. James et al. suggest age is the strongest predictor of GI pathology [2]. Annibale et al. concluded that age and gender, not Hb or IDA duration, were the strongest predictors [14]. We found no significant relationship between the age and the GI endoscopic finding in general, nor for malignant GI lesions in particular.

The lower and upper GI endoscopies were negative in 118/296 and 25/127 patients, respectively, (with the addition of the 25 patients who had BDE for each group). Additionally, there were 121 patients with simple nonspecific gastritis or nonspecific inflammatory colonic changes. Niv et al. documented no GI endoscopic changes in approximately 29% of asymptomatic patients [4]. One study suggested asymptomatic IDA patients with normal endoscopy findings have excellent prognoses [13].

Rocky and Cello identified a potential cause of IDA in 62/100 patients, with 36% of patients having upper GI pathology and found that localizing symptoms to either the upper or lower GI tract were predictive for the disease [20]. In contrast, Wilcox et al. uniquely focused on 52 asymptomatic patients with IDA and tried to build criteria for diagnostic priorities [13].

Although atrophic gastritis (AG) infrequently causes IDA, many studies included AG as a significant GI lesion in IDA patients [4,7,15]. The hypochlorhydria that may accompany AG can impair or inhibit iron absorption [20]. In this study, 31 patients (i.e., 24% of patients who had esophagogastroduodenoscopy(EGD)/7.8% of all asymptomatic patients) had AG. This proportion aligns with those reported by several other studies (range, 2.3% to 23.2%) [7,15]. The yields of patients in this study who had AG was larger than that reported by Niv et al. (14.5%) [4] and less than that reported by Marignani et al. (50%) [21].

Around 6% of all asymptomatic patients and 20% of the patients who underwent EGD had peptic ulcers, findings similar to those reported in other studies (range, 2% to 17%) [1,5,12,14,15]. We could not verify any significant association between IDA severity and the presence of peptic ulcer as ascertained by Kim et al. [1] because the ulcer tends to be symptomatic, not asymptomatic [13], causing the low percentage.

CD in asymptomatic IDA patients is called atypical or “silent.” Twelve percent of asymptomatic patients with EGD (3.8% of the total enrolled patients) had CD, similar to the percentages reported in previous studies (2.9% to 6%) [3,7,19].

The clinical spectrum of CD is broad and includes a classic presentation of malabsorption with diarrhea, no classical extra-intestinal features, subclinical or asymptomatic more common silent forms, and potential disease characterized by positive serology with a healthy intestinal mucosa on biopsy [8]. Advancements in serologic testing have dramatically changed the epidemiology of CD, worldwide, by revealing the higher incidences of silent or atypical CD [11], with an associated decreased quality of life, and increased mortality [22].

Although previous reports revealed a lack of correlation of the degree of villous atrophy with the severity of the clinical presentation of CD [23], we could not evaluate this statistically due to the low number of cases of biopsy-proven CD patients (n=15). 

Individuals with asymptomatic CD do not manifest the symptoms commonly associated with CD, and their response to gluten withdrawal is different than expected. These patients are often diagnosed accidentally through population screening, or during the case-detection strategies in high-risk patients [8]. They are also diagnosed during small bowel biopsy for other investigations, especially when diarrhea is absent [13].

IDA may indicate the presence of possible GI malignancies, and an inadequate evaluation may cause an unnecessary diagnostic delay [7]. The current guidelines indicated colonoscopy in the screening of asymptomatic, average-risk patients for CRC [3]. We found no significant association between GI malignancy diagnosis with patient age, gender, or IDA severity for the 26 asymptomatic patients with GI malignancies (Table 2).

The endoscopic diagnostic yield for GI malignancies was 6.53% (26/398 patients). Asymptomatic GI malignancies may present with IDA, and seeking these conditions is a top priority. Previous studies report a malignancy detection rate of 5.3% to 6.5% [3,12]. Still, the highly cited studies by Wilcox, Annibale, and Bini et al. [13,14,24] report malignancy detection rates of 3% to 21%, but they considered these figures as non-representative.

Although malignant tumors may occur in asymptomatic premenopausal women, such findings are rare. Our study had only one case of upper and one case of lower GI tumors detected in asymptomatic premenopausal women, and this rate of detection is similar to the guideline consensus [3].

Other studies concluded that the GI malignancies are more common in asymptomatic patients with IDA and recommend referral for GI evaluation [25]. Niv et al. reported a high rate of colon malignancies-predominantly right-sided colon carcinoma in older patients with asymptomatic IDA [4]. Van Mook et al. reported that EGD should always be performed in asymptomatic patients with IDA and negative colonoscopies, certainly among elderly patients and NSAIDs users, even though upper GI carcinoma will probably be an infrequent finding [17]. Dignass et al. found a good correlation between activity, severity, and the amount of blood loss in intestinal neoplasia [26].

Twenty-nine patients had non-bleeding hemorrhoids (7.3% of the total asymptomatic IDA patients) as a potential cause of IDA. Park et al. [15] demonstrated that non-bleeding hemorrhoids constitute 15.7% of the patients in anemic young women who lack GI symptoms.

The colonoscopy revealed that nine patients had IBD (i.e., 3%). IDA can be the critical early manifestation of Crohn’s disease. Crohn’s disease was diagnosed in one asymptomatic woman with IDA by Annibale et al. during their thorough examination of 71 patients with IDA [14]. A rapid recurrence of IDA in asymptomatic patients should raise suspicions of subclinical inflammatory activity [26].

Eight of 25 patients had dual lesions discovered in BDE. Fireman et al. suggested BDE use as part of the workup for IDA in most of the asymptomatic patients with IDA and recommended lower GI endoscopy first [27]. Rockey and Cello had reported one woman with asymptomatic IDA had dual lesions found during BDE [20]. Serefhanoglu et al. recommended BDE in the initial diagnostic sequence for IDA, particularly in men older than age 50 and in high-risk postmenopausal women with suspected occult bleeding lesions [7].

Both American and European guidelines for the evaluation and screening of asymptomatic IDA recommend synchronous bundling of colonoscopy and EGD in an arbitrary order [3,28], while Stephens et al. favored investigating the lower GI tract first, or performing both EGD and colonoscopy during the same session [29].

We found no correlation between the gender, age, or any IDA parameter with the diagnosis of GI malignancies. Yet, the older patients with atypical symptoms or higher suspicion of CRC are probably over presented because of the quick referral for evaluation.

Our study was limited in that it was hospital-based at three medical centers in a single city. The drug history for the asymptomatic inpatients was scarce, and we had no comparison to symptomatic IDA patients. Finally, the database created for this study did not track the outcomes in patients found to have cancer at endoscopy, especially those patients diagnosed with cancer who subsequently had surgical treatment.

However, our large sample size and high diagnostic yield strengthen the study. A possible explanation for the high endoscopic yield in this patient population was our study took place in hospitals rather than outpatient settings. Previous studies investigated less homogeneous groups of patients, composed of both referred inpatients and outpatients [16,20]. Moreover, most previous studies involved older patients with a mean age ranging from 60 [20] to 70 years [18].

## Conclusions

The presence of symptoms is of limited value in guiding the diagnostic procedure concerning GI etiology. Asymptomatic patients with IDA should undergo endoscopic examination, irrespective of the IDA parameters, age, or gender. Asymptomatic IDA obligates a comprehensive and thorough GI examination, as a large proportion of patients may have potentially treatable pathologies. Also, the non-verified significance between the GI malignancy and the IDA severity merit further studies on the effectiveness of the evaluation, including its cost-effectiveness. The findings of different GI malignancies necessitate prompt exhaustive surveillance of this group of patients, given their risk of malignancy, especially in severe IDA cases. Studies collecting the results of colonoscopies of different age groups would further benefit gastroenterologists and provide data that may prompt changes in the current treatment guidelines.
